# Alstrom syndrome (OMIM 203800): a case report and literature review

**DOI:** 10.1186/1750-1172-2-49

**Published:** 2007-12-21

**Authors:** Tisha Joy, Henian Cao, Graeme Black, Rayaz Malik, Valentine Charlton-Menys, Robert A Hegele, Paul N Durrington

**Affiliations:** 1 Department of Vascular Biology and Medicine, Robarts Research Institute and Schulich School of Medicine and Dentistry, University of Western Ontario, London, Ontario, Canada; 2 Clinical and Laboratory Sciences, Medical Genetics, Eye Hospital, Manchester, UK; 3 Cardiovascular Research Group, School of Clinical & Laboratory Sciences, Core Technology Facility (3^rd ^Floor), Manchester, UK

## Abstract

**Background:**

Alstrom syndrome (AS) is a rare autosomal recessive disease characterized by multiorgan dysfunction. The key features are childhood obesity, blindness due to congenital retinal dystrophy, and sensorineural hearing loss. Associated endocrinologic features include hyperinsulinemia, early-onset type 2 diabetes, and hypertriglyceridemia. Thus, AS shares several features with the common metabolic syndrome, namely obesity, hyperinsulinemia, and hypertriglyceridemia. Mutations in the *ALMS1 *gene have been found to be causative for AS with a total of 79 disease-causing mutations having been described.

**Case presentation:**

We describe the case of a 27-year old female from an English (Caucasian) kindred. She had been initially referred for hypertriglyceridemia, but demonstrated other features suggestive of AS, including blindness, obesity, type 2 diabetes, renal dysfunction, and hypertension. DNA analysis revealed that she is a compound heterozygote with two novel mutations in the *ALMS1 *gene – H3882Y and V424I. Examination of her family revealed that her phenotypically unaffected mother and younger sister also had heterozygous mutations in the *ALMS1 *gene. In addition to presenting these novel molecular findings for AS, we review the clinical and genetic features of AS in the context of our case.

**Conclusion:**

Two novel mutations in the *ALMS1 *gene causative for AS have been reported here, thereby increasing the number of reported mutations to 81 and providing a wider basis for mutational screening among affected individuals.

## Background

Alstrom syndrome (AS; OMIM 203800) was first described in 1959 and has an estimated prevalence of <1:100 000 [1,2]. AS is an autosomal recessive multiorgan disorder, characterized by childhood obesity, adult short stature with initial accelerated childhood linear growth, progressive cone-rod dystrophy leading to blindness, and sensorineural hearing loss [3,4]. Endocrinologic complications include early-onset diabetes mellitus (typically in the 2^nd ^or 3^rd ^decades), hyperinsulinemia (with associated acanthosis nigricans), hypertriglyceridemia, infertility (hypergonadotrophic hypogonadism), and hypothyroidism [4-6]. Systemic fibrosis is commonly observed [3]. The primary cause of mortality among young affected patients is cardiac involvement from dilated cardiomyopathy whereas renal failure is the major cause of death among the older subgroup [2,3].

Mutations in the *ALMS1 *gene were independently identified as causative for AS by two research groups [7,8]. *ALMS1 *encodes a protein of 4169 amino acids, which includes a large tandem-repeat domain consisting of 47 amino acids (aa); the exact function of the ALMS1 protein still remains unknown [7]. However, the ALMS1 protein has been shown to be ubiquitously expressed and to localize subcellularly [9]. It has been proposed that ALMS1 is involved in the functioning of centrosomes or basal bodies [9]. Although initial data revealed normal ciliary structure in fibroblasts from affected individuals with *ALMS1 *mutations, *ALMS1 *knockout mice demonstrated abnormal ciliary structure that could be rescued with a prematurely truncated fragment of ALMS1 containing the N-terminus [9,10]. Thus, the N-terminus of ALMS1 seems to be crucial to normal ciliary structure [10]. To date, a total of 79 disease-causing *ALMS1 *mutations have been reported [11]. We report here the clinical and novel molecular findings in a Caucasian kindred with Alstrom syndrome from the United Kingdom and review the current clinical and molecular genetic aspects of this condition.

## Case presentation

In 2002, the 27-year old proband was referred to the lipid clinic of a tertiary health care centre for evaluation of an elevated triglyceride (TG) level of 59.1 mmol/L. Her prior history included poor vision since birth, commencing with the development of night blindness, eventually resulting in legal blindness by the age of 17. She had undergone a left nephrectomy at the age of 24 for a perinephric abscess due to chronic pyelonephritis. Ultrasound evaluation revealed a normal-sized right kidney with evidence of cortical scarring. Hypertension and diabetes subsequently developed at the ages of 25 and 26 years, respectively. She experienced learning difficulties in school, but did not have sensorineural deafness. On physical examination, there was evidence of central obesity with her body mass index (BMI) being 34.9 kg/m^2^. Her blood pressure on antihypertensive treatment was 132/86 with a regular pulse of 80 beats per minute. There was no evidence of poly- or syndactyly suggestive of Bardet-Biedl syndrome. Hirsutism was present on the face, abdomen, and arms. Ophthalmologic examination was notable for retinitis pigmentosa and cataracts bilaterally.

**Figure 1:**
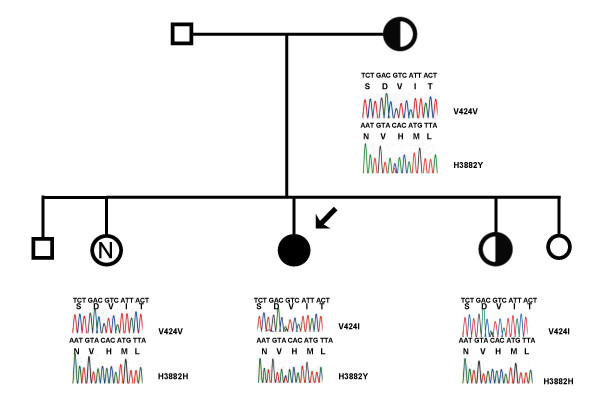
**DNA sequence analysis of Alstrom syndrome mutations**. Proband is indicated by the solid circle and arrow. Smaller symbols represent individuals from whom DNA was unavailable. There is no known consanguinity between the parents of this kindred. Electrophoretic tracings for exons 6 and 17 of the *ALMS1 *gene from the proband and available immediate relatives are shown. The proband is a compound heterozygote for the V424I and H3882 Y mutations while the mother demonstrates heterozygosity only for the H3882Y mutation and the younger sister demonstrates heterozygosity only for the V424I mutation. The older sister has no mutations in the *ALMS1 *gene.

Her family consisted of non-consanguineous parents, both alive and well, as well as four siblings – three sisters (aged 18, 26, 29 years) and one brother (aged 29 years), who were also healthy. The family structure is outlined in Figure 1.

### Biochemical attributes and investigations

The proband's initial labwork at the time of consultation revealed a plasma TG level of 20.6 mmol/L with a high-density lipoprotein cholesterol (HDL-C) level of 0.56 mmol/L. Her fasting glucose and insulin levels on oral antidiabetic therapy but not insulin were 14.2 mmol/L and 68.1 mU/L (normal <10 mU/L), respectively, with a HbA1C of 11.2%. Her creatinine was elevated at 255 **μ**mol/L with overt proteinuria of 2.68 g/24 hours. Her total alkaline phosphatase of 416 U/L (normal < 330 U/L) demonstrated increases in both the liver and bone isoenzymes. Abdominal ultrasound revealed fatty infiltration of the liver and splenic enlargement (14 cm in length). Both her electrocardiogram and echocardiogram were normal.

The results of lipoprotein analyses of the proband, proband's mother and two sisters are shown in Table 1. The proband demonstrated significant hypertriglyceridemia (TG = 20.6 mmol/L) and hypoalphalipoproteinemia (HDL-C = 0.56 mmol/L). Both the proband's mother and older sister displayed mildly elevated plasma TG whereas the younger sister tested had relatively normal plasma TG. Plasma low density lipoprotein-cholesterol (LDL-C) levels were higher among the mother and younger sister compared to the others. The proband demonstrated the lowest LDL-C and HDL-C levels of all four members of the kindred examined.

**Table 1:** Clinical and biochemical characteristics of the pedigree

**Biochemical parameter**	**Proband (II-2)**	**Mother (I-1)**	**Sister (II-1)**	**Sister (II-3)**
Total cholesterol (mmol/L)	7.40	7.70	5.78	6.79
Triglyceride (mmol/L)	20.6	2.61	3.80	1.78
VLDL- C (mmol/L)	4.62	0.92	1.09	0.58
VLDL-TG (mmol/L)	15.4	1.57	2.54	1.10
VLDL-TG/VLDL-C ratio	3.33	1.71	2.33	1.90
HDL-C (mmol/L)	0.56	0.94	1.04	1.72
LDL-C (mmol/L)	0.83	5.32	3.29	4.19
Apo A-1 (g/L)	1.25	1.20	1.45	1.59
Apo B (g/L)	0.91	1.59	1.16	1.22

Abbreviations: VLDL-C, very low density lipoprotein – cholesterol; VLDL-TG, very low density lipoprotein- triglyceride; HDL-C, high density lipoprotein – cholesterol; LDL-C, low density lipoprotein – cholesterol

### Treatment

Treatment of the proband's hypertriglyceridemia required the use of multiple therapies, including a low-fat diet (< 20 g fat per day), insulin (glargine 14 units/day), pioglitazone (45 mg/day), atorvastatin (40 mg/day), and omega-3-acid ethylesters (Omacor) (2 g bid). As a result of therapy, TG levels decreased by ~84% to 3.30 mmol/L and HDL-C levels improved by ~252% to 1.41 mmol/L.

### DNA isolation and sequence analysis

Informed consent was obtained from all available subjects or their guardians, and the study was approved by the institutional review board of the University of Western Ontario (#07920E). DNA and lipoprotein analyses were unavailable for the father, one sister (aged 18 years), and brother (aged 29 years). Genomic DNA from the four available study subjects was isolated from whole blood (Puregene, Gentra Systems, Minneapolis, MN). Exons 1 to 23 of *ALMS1 *were amplified using the primers in Table 2. The final volume of 50 **μ**L contained 32 pmol of each primer, 0.2 mM each of dATP, dCTP, dGTP, and dTTP, 1.5 mM MgCl_2_, 50 mM KCl, 20 mM Tris-HCl (pH 8.4), and 2.5 units of Taq platinum DNA polymerase (Life Technologies, Mississauga, Ontario, Canada). DNA amplifications were performed with denaturing at 94°C for 5 minutes, followed by 30 cycles of a denaturing step at 94°C, an annealing step at 60°C, and an extension step at 72°C, each for 30 seconds. A final extension step at 72°C was performed for 10 min. Amplification products were electrophoresed on 1.5% agarose gels and purified with the QIAEX II gel extraction kit (Qiagen, Inc., Mississauga, Ontario, Canada). Purified DNA fragments were sequenced by the chain termination method using the ABI 3730 Automated DNA Sequencer and analyzed using Sequence Navigator Software (both from PE-Applied Biosystems, Mississauga, Ontario, Canada).

**Table 2:** Oligonucleotides for genomic amplification and sequencing of *ALMS1*

**Exon**	**Forward Primer**	**Reverse Primer**
1	GCACTGCGCCTAAGCTG	CAGCCTCCACCCCCAAC
2	ATGTGAAAGGGCTTTATAAACTGG	TTTTTCCATTCTTCATAGCTAAATCA
3	CAGTTAATGACTTAGCATGTTTTCCT	TCCTTAACTCAAAAAGGGGAAAG
4	ACGTAAGTAAATAATCAATTTTCAGCA	TCTAAGCCCCACCTCAAAGT
5	TTTCAGTGACATATGTATTTTTGTGTT	TTCCCTTGGGAATTTTATTTTT
6	CTTCGTGTGTGGGAGCTGAG	CAATACTGAAAAAGGCCACGTT
7	TGGGCATTAATGAGTCTTTTTC	TTTTCACAAGGTATCCGTAAGTAGG
8	GCTTTTTAAAGGCTCAAAGCTG	TCTCTCTATGTGAGTAGGAAGTAGAGG
8	TGACCAGACAACTGGCATGT	GACTGTCTGCTAAGTCCTGTGG
8	TTCTTACTCACAAAGAGAAAAGCCTA	GGGCAGCCAATACAGAAACA
8	TTTCCCTGAAGAAGCTCTGAA	TGGCAAGGTCTGTTGGTAGA
8	TCACAAAGAGAGAAGCCTGGT	AGCTGGTGTGCCAGTTGTCT
8	TTCAGTTGCCTCTGAACCAG	TGTGGCAAGACCTGTTGGTA
8	CACACACAGAGAAGCCTGGT	AAAGGTCCTGCTGGTATGTCA
8	TCCATTGTTTCTGGACCTACTG	ATCTGGCAACTCTTGCTGGT
8	ACTGTAACTTCCTCTTTCTATTCACAT	TCTCAGTCTTCCGGTCACCT
8	AGCAGGAGTTGCCAGATGTT	CTGGTTTTCCAGTATTCACATCA
8	AAAGATTTCAGCTGTCCCTGA	CTGCATCCTGGATTTCTTCA
8	CTCAGGCTGATGACAGAGTTG	CCCAATGGTTCCACTACACC
8	GAGCAAAGTCAGTATGGCATTAGA	TGGCTAAGCTTCCTCAAAACA
9	TCTTCTGTGTTGCAATTGTTGA	TTCCATCACCCATTCTTTCA
10	TTGGACTACTTCAAATAAGAACCTG	GACGGCATTTGTGATGAAGA
10	ACCTGCTTTTGTGCCACCTA	CTTGGTCTGCCCATGCTAAT
10	CCAGTACCAGGGCAAATTGT	GGAAGGGGAAAATGGTGTTT
10	ACCTTCCGTCTCCCATTTCT	TCCTGTGCTACAGGTTTACTGG
10	GCTTCTAAAGCGAGGATGAA	CCCCCAAGAACCGATATCTA
11	TTCCTTGAAACCACTTTTGGA	GAAAGACACAACCACAAATTTCTAA
12	GAAGGCATTCCATATTTGTTCA	GCACTGGACTTTTGTCACTCC
13	TCATAGAATTGGTCTAAGAGGCAAA	AAGATTGGATAGTAATCTCATTTAGGA
14	ATGGGTTTGGGGTTTTGTTT	GAGCTGAAGACAGCAAGAAGAA
15	AACAAAGCCTTTCACATAATACG	CACTGACCCTCACATACACAC
16	GCAGGCAGTGAATTTTCTGAT	TTTTGGATAATCTCTAACTTGACTTTT
16	CCAGAATAAAGAGCCTCAGCA	TTTTTAAGCTCGCCTGTATTTTT
16	GCGGTTTAAAAGCCTAGAGAAA	TTTTCACCTGTGTGCAAAGC
17	TGAATTGGATTAGAAAGAGGACTTG	TCTTACATGTTTAAGAGCCATTTCA
18	TCCCACACAAAGGGATTGTA	ATCGCAGGGGACTTGAAAT
19	CTGGGTGGGGCTGTAAAAA	CCAAGTCACAGAGCCAGCTT
20	GCATATGGAGAGTAGATTGCATCA	TGGGCTGGCCTTTAGCAG
21	GGTAGGGGCACCAAGTCCTA	CAGAGCTCCCGACCACTTG
22	GATGAGCTCCTGGAGAGTGG	GGCAACGTGTTTTCTCCATT
23	GGCATCTGCCTCTGATGG	AAGGATTCTGCTTCTCTAGGTTCA

### Identification of novel disease-causing *ALMS1 *mutation

Sequencing of genomic DNA from the affected proband demonstrated no mutations in the coding regions of the *LPL *gene (data not shown), but did reveal 2 novel missense mutations in the *ALMS1 *gene – a G→A transversion at nucleotide 1381 of exon 6 and a C→T transversion at nucleotide 11755 in exon 17. These mutations resulted in the replacement of valine by isoleucine at codon 424 (V424I, exon 6) and histidine by tyrosine at codon 3882 (H3882Y, exon 17). The proband's mother was heterozygous for the H3882Y mutation while the proband's younger sister was heterozygous for the V424I mutation, proving that the mutations were on opposite chromosomes in the proband. The proband's older sister was unaffected both clinically and molecularly. See Figure 1.

The missense mutation V424I was subsequently genotyped in 200 healthy unaffected Caucasian controls using the primer pair and PCR conditions for exon 6 amplification. The amplified product was digested with restriction enzyme *Aat*II (New England Biolabs Inc., Ipswich, MA, USA). All healthy controls were homozygous for G at nucleotide 1381. Similarly, the missense mutation H3882Y was genotyped in 200 healthy unaffected Caucasian controls using the primer pair and PCR conditions for exon 17 amplification. The amplified product was digested using the restriction enzyme *Rsa*I (New England Biolabs Inc., Ipswich, MA, USA). Among the 200 controls, only 1 control was found to be heterozygous at nucleotide 11755 C/T, giving an allele frequency of 0.9975 for the C allele and 0.0025 for the T allele at this position, based on the Hardy-Weinberg equation. This suggests that the second heterozygote mutation, 11755 C→T, is present in the general population, albeit at a very low frequency.

## Literature review and case discussion

The distribution of AS is global without any gender predilection. With an estimated prevalence of < 1:100 000, only ~500 cases of AS have been reported in the literature thus far [2-4,7,11-37]. Yet, a greater proportion of kindreds of English descent have been noted in the literature. Whether this is due to selection bias from kindreds in the United Kingdom or North America having more readily available health care access compared to kindreds from developing nations is uncertain. Certainly, awareness of AS is lacking despite the complexity and potential lethality of this disorder. Moreover, in addition to childhood obesity, affected individuals may develop insulin resistance, type 2 diabetes, and hypertriglyceridemia. Thus, AS can be thought of as a rare genetic disorder with several features similar to the common metabolic syndrome.

### General clinical features

Prior to the recent discovery of *ALMS1 *mutations causative for AS, the diagnosis of AS was made solely based on phenotype. However, AS exhibits a great degree of phenotypic variability, even within families, thereby creating difficulties for a universal definition of AS [17,18]. Recently, Marshall et al defined AS using age-specific criteria [38]. See Table 3.

**Table 3:** Diagnostic criteria for Alstrom syndrome [38]

**Age (years)**	**Major Criteria**	**Minor Criteria**	**Other supportive evidence**	**Diagnosis**
≤ 2*	• *ALMS *1 mutation in 1 allele and/or family history of AS • Vision (nystagmus, photophobia)	• Obesity • DCM/CHF	• Recurrent pulmonary infections • Normal digits • Delayed developmental milestones	2 major criteria OR 1 major + 2 minor criteria
3–14	• *ALMS *1 mutation in 1 allele and/or family history of AS • Vision (nystagmus, photophobia, decreased acuity, cone dystrophy by ERG**)	• Obesity and/or insulin resistance • (History of) DCM/CHF • Hearing loss • Advanced bone age • Hepatic dysfunction • Renal failure	• Recurrent pulmonary infections • Normal digits • Delayed developmental milestones • Hyperlipidemia • Scoliosis • Flat wide feet • Hypothyroidism • Hypertension • GH deficiency • Recurrent UTI	2 major criteria OR 1 major + 3 minor criteria
≥ 15	• *ALMS *1 mutation in 1 allele and/or family history of AS • Vision (legal blindness, history of nystagmus in infancy/childhood, cone and rod dystrophy by ERG)	• Obesity and/or insulin resistance and/or DM2 • (History of) DCM/CHF • Hearing loss • Hepatic dysfunction • Renal failure • Short stature • Males – hypogonadism • Females – irregular menses and/or hyperandrogenism	• Recurrent pulmonary infections • Normal digits • History of developmental delay • Hyperlipidemia • Scoliosis • Flat wide feet • Hypothyroidism • Hypertension • GH deficiency • Alopecia • Recurrent UTI or urinary dysfunction	2 major + 2 minor criteria OR 1 major + 4 minor criteria

*These diagnostic criteria should be re-evaluated when the patient becomes older

** ERG to be conducted only if the child is old enough for testing

Abbreviations: AS, Alstrom syndrome; DCM/CHF, dilated cardiomyopathy/congestive heart failure; ERG, electroretinogram; GH, growth hormone; UTI, urinary tract infections; DM2, type 2 diabetes.

### Neurosensory and cognitive features

Sensorineural hearing loss and congenital retinal dystrophy are two cardinal features of AS. In fact, pendular or searching nystagmus and photodysphoria are often evident before the age of 1 yr. There is initial loss of cone function followed by rod disintegration, ultimately leading to early blindness. By the age of 16, ~90% of affected individuals are blind [38]. In addition, development of subcapsular cataracts may contribute to vision loss [3,6]. Exudative retinopathy has also been reported in AS [12]. Meanwhile, ~80 % of affected individuals will develop bilateral sensorineural hearing loss [3]. This usually occurs at a later age in childhood and is characterized by the initial loss of high frequency sounds. Progressive deterioration in hearing occurs, and is sometimes accompanied by conductive hearing loss due to chronic otitis media or glue ear [3]. These early changes in neurosensory capabilities have tremendous impact not only on the social development of the child but also on his/her adaptation to the external environment.

Although delay of cognitive development is not a common feature of AS, delay in developmental milestones is seen in ~45% of AS-affected children. Other neurologic manifestations may include absence seizures and general sleep disturbances [3]. The frequency of mood and psychiatric disorders in AS-affected individuals has not been determined.

### Anthropometric and growth measures

Individuals with AS often have distinctive facial features, such as round face, deep-set eyes, thick ears, dental anomalies, hyperostosis frontalis interna, and premature frontal balding. Their toes and fingers are typically short and stubby with no polydactyly or syndactyly while their feet are typically noted to be wide and thick [38].

Childhood obesity is present in over 95% of individuals with AS [3]. However, both waist circumference and body fat percentage (as measured using dual-energy X-ray absorptiometry) negatively correlated with age, independent of BMI, indicating the possible recruitment of more metabolically active fat stores [2]. The presence of hyperphagia has been controversial [3,4]. Although childhood is often accompanied by rapid growth with height above the 50^th ^percentile before puberty, most individuals demonstrate a decreased adult height [2,3]. This height discrepancy is supported by evidence for advancement of the bone age by 1–3 years prior to puberty as described by Marshall et al [3]. As well, abnormalities of the insulin growth factor (IGF) system of affected patients have been demonstrated [13]. Yet, the exact reasons for short stature remain to be determined.

### Endocrinologic features

Similar to the features of the metabolic syndrome seen in the general population, patients with AS also demonstrate hypertriglyceridemia and type 2 diabetes. Type 2 diabetes is diagnosed in over 80% of individuals above the age of 16 while insulin resistance and hyperinsulinemia have been demonstrated in individuals as young as 1 year old, even prior to the onset of obesity [3,38]. Meanwhile, hypertriglyceridemia is seen in ~50% of affected individuals, with acute pancreatitis secondary to hypertriglyceridemia occurring in ~5% [3]. Other endocrinologic manifestations of AS include hypothyroidism, hypogonadism (particularly among men), alterations in the onset of puberty, ovarian cysts and hirsutism (among females), and short stature with abnormalities in the IGF-growth hormone system [3,13].

### Cardiorespiratory features

Dilated cardiomyopathy (DCM), affecting ~60% of individuals, can occur at any age, but most typically during infancy. Although DCM is the most common underlying cause of death in the infantile period, survival with infantile-onset of DCM tends to be better than that for adult-onset DCM. Marshall et al showed that while one-third of adult-onset DCM patients died, ~74% of infantile-onset DCM patients survived [3]. However, children and adults who have survived infantile-onset DCM are still susceptible to sudden recurrences [38]. In addition to cardiac problems, AS-affected patients can have a variety of respiratory problems, including chronic asthma, sinusitis/bronchitis, alveolar hypoventilation and recurrent pneumonia [3].

### Gastrointestinal and genitourinary involvement

Hepatic involvement in AS was first described in 1991 [39]. Approximately 80% of patients affected with AS may have hepatic involvement, ranging from mild elevation in liver transaminases to hepatic steatosis to overt cirrhosis with portal hypertension [3,30,31]. Other gastrointestinal effects include upper gastrointestinal pain, chronic diarrhea, constipation, and gastroesophageal reflux [3].

Renal insufficiency occurs in ~50% of AS-affected individuals. Whether hypertension is a consequence of renal insufficiency or contributes to renal insufficiency is uncertain, but it is present in ~30% of individuals [3]. Urologic dysfunction is common in both men and women and can be manifest as urge incontinence, poor flow, urinary retention, or difficulty initiating voiding [3]. Urologic anatomical abnormalities can also occur in AS, including calyceal deformities, narrowed ureteropelvic angles, dilated ureters, and misalignment of the kidneys [6].

### Differential diagnosis

It remains important to distinguish AS from other disorders characterized by childhood obesity and retinal dystrophy, such as the Laurence-Moon and Bardet-Biedl syndromes. Normal mentation and lack of poly/syndactyly help to distinguish AS from Bardet-Biedl, while deafness and the absence of spastic paraparesis help to differentiate AS from Laurence-Moon. Refsum disease is a rare disorder characterized by hearing loss, visual loss, and hepatic involvement, but also includes several features not associated with AS [40]. See Table 4 for clinical features of these disorders.

**Table 4:** Differential diagnosis for Alstrom syndrome [41, 42]

	**Alstrom syndrome**	**Laurence- Moon syndrome**	**Bardet- Biedl syndrome**	**Refsum disease**
Inheritance	AR	AR	AR	AR
Childhood Obesity	+	+	+	-
Visual Impairment	+	+	+	+
Sensorineural Deafness	+	-	-	+
Short Stature	+	-	-/+	-
Diabetes Mellitus	+	-	+	-
Renal Disease	+	-	+	-
Polydactyly/syndactyly	-	-	+	+
Mental Delay	-	+	+	+/-
Hypogonadism	+	+	+	-
Dilated Cardiomyopathy	+	-	-/+	-/+
Other	Hepatic Involvement	Spastic paraplegia	-	Anosmia Dysmorphic features Cerebellar ataxia Polyneuropathy

Abbreviations: AR, autosomal recessive

### Genetic features of AS

**Figure 2:**
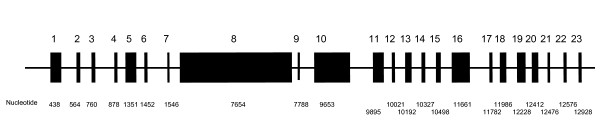
Normal *ALMS1 *gene structure*. * Figure 2 is not drawn to scale.

**Figure 3:**
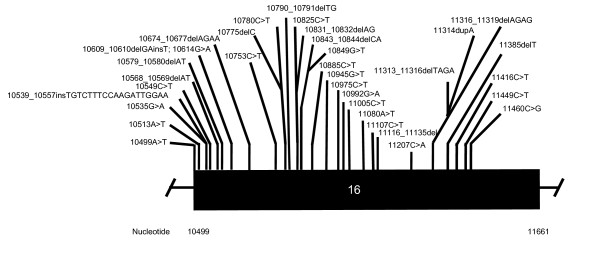
Reported *ALMS1 *Mutations for Exon 16*. * Figure 3 is not drawn to scale.

**Figure 4:**
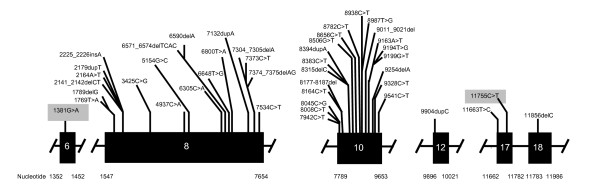
Reported *ALMS1 *Mutations for Exons 6, 8, 10, 12, 17, 18*. * Figure 4 is not drawn to scale. The two novel mutations found in this study are shaded in gray. Other mutations not included in the figure are AC074008.5:g.124455_125899del and 10483C>T.

AS is an autosomal-recessive disease that demonstrates intrafamilial and interfamilial variation. To date, a total of 81 disease-causing *ALMS1 *mutations have been reported, including the 2 reported from our group [11]. A schematic representation of the normal *ALMS1 *gene and the *ALMS1 *mutations causative of AS are shown in Figures 2, 3, 4.

Affected individuals of most kindreds demonstrate compound heterozygosity with the majority of *ALMS1 *mutations reported being nonsense. Approximately 40% of all known *ALMS1 *mutations occur in exon 16 while other *ALMS1 *mutational hotspots include exon 10 (23%) and exon 8 (21%) [11]. Mutations in exon 16 have correlated with a more severe disease phenotype, manifest as early onset of retinal degeneration before the age of 1 year (p = 0.02), presence of urologic dysfunction (p = 0.02), occurrence of dilated cardiomyopathy (p = 0.03), and development of diabetes (p = 0.03). Meanwhile, the severity of renal disease in AS has correlated with mutations in exon 8 [11].

Among 250 AS-affected individuals examined, 15 synonymous single nucleotide polymorphisms (SNPs), 51 nonsynonymous SNPs, and a deletion of 3 nucleotides in exon 8 have also been described. Using prediction modeling, Marshall et al have determined that 8 of these variants are disease-causing [11]. The most common *ALMS1 *mutation is c.10775delC, which is observed in 50% of reported English kindreds. Similarly, haplotype sharing has been demonstrated amongst members of Turkish kindreds, indicating the possibility of separate founder effects in both the English and the Turkish kindreds [11].

### Discussion of our case

Our results indicate that *ALMS1 *mutations of V424I and H3882Y are the molecular basis for the Alstrom phenotype in the proband presented here. The H3882Y mutation was present in only 1 of 200 normal healthy controls while the V424I mutation was absent in all 200 controls. These two disease-causing mutations have not been reported before and represent the first mutation reported for exon 6 and the second reported for exon 17 [11]. Interestingly, the predicted frequency of homozygotes for the H3882Y mutation would be 1 in 160 000, signifying that, if homozygotes are affected, AS may indeed be more prevalent than previously thought.

To date, with the 2 missense mutations of our kindred, there are a total of 10 missense AS-causing mutations. Marshall et al showed that the majority of kindreds affected by AS, as in our kindred, demonstrate compound heterozygote mutations [11]. The V424I mutation localizes to the N-terminus of the ALMS1 protein while the H3882Y mutation localizes to the C-terminus of the protein [7]. Since even fragments of the N-terminus have been shown to be able to normalize the abnormal ciliary structure of *ALMS1 *knockout mice, it would be expected that isolated heterozygous mutations still including the relatively intact N-terminus would not necessarily result in significant clinical findings, as evidenced in our proband's mother and younger sister [10]. The functional importance of the C-terminus remains to be determined. Importantly, no correlations could be drawn between the presence of a single *ALMS1 *mutation, as seen in the mother and younger sister, and abnormalities in any of the lipid parameters examined.

Hypertriglyceridemia was evident in our proband and is a common finding among individuals affected with AS. Our proband had no documented episodes of pancreatitis, but was at high risk for pancreatitis given her significant hypertriglyceridemia. There can be multiple factors contributing to hypertriglyceridemia among AS-affected patients, including insulin resistance or diabetes, liver dysfunction, renal dysfunction, dietary factors, and alcohol intake. However, examination of a group of AS-affected individuals demonstrated no significant correlations between hepatic or renal function, BMI, or degree of glucose intolerance and high TG levels. Instead, there was indeed a major correlation between hyperinsulinemia and hypertriglyceridemia in that study [5]. Our proband demonstrated significant hypertriglyceridemia, accompanied by insulin resistance (type 2 diabetes), elevated BMI, and renal dysfunction. A multifaceted treatment approach resulted in improvements in TG and HDL-C to 3.30 mmol/L and 1.41 mmol/L, respectively, demonstrating that it is possible to effectively treat lipid abnormalities among AS-affected individuals.

The correlation of mutations in exons 6 or 17 to phenotype remains to be determined. Interestingly, our proband did not have sensorineural deafness, which typically occurs in ~84% of affected individuals [11]. No genotype-phenotype correlations have been made for sensorineural deafness [11]. However, it is possible that mutations in exon 6 or 17 may not be associated with sensorineural deafness although further kindreds with these mutations are required to substantiate this possibility. Yet, the mutations in this proband seem to have resulted in an "intermediate-severity" phenotype as judged by an early onset of retinal degeneration, severe renal dysfunction, and significant elevation of TG levels, but a later development of diabetes and a lack of dilated cardiomyopathy.

## Conclusion

We have reported here two novel missense mutations in the *ALMS1 *gene causative for Alstrom syndrome in an English kindred. These extend the mutational spectrum in AS and provide a resource for mutational screening. Furthermore, we hope that these mutations may eventually add insight into the function of the ALMS1 protein and contribute to the understanding of the phenotypic variety observed among AS-affected individuals.

## Competing interests

The author(s) declare that they have no competing interests.

## Authors' contributions

TJ wrote the manuscript. HC participated in the design of the study and carried out all the molecular genetic studies for the family. GB examined the proband and suggested the diagnosis of AS in the proband, which resulted in molecular genetic testing. RM played a crucial role in the management of the proband. VC-M and PND conducted the examination of the proband and her family, obtained consent for molecular genetic testing, and drafted the manuscript. RAH conceived the design of the study and provided critical revisions to the manuscript. All authors read and approved the final manuscript.
